# The effect of acute kidney injury on long-term health-related quality of life: a prospective follow-up study

**DOI:** 10.1186/cc12491

**Published:** 2013-01-28

**Authors:** José GM Hofhuis, Henk F van Stel, Augustinus JP Schrijvers, Johannes H Rommes, Peter E Spronk

**Affiliations:** 1 Department of Intensive Care, Gelre Hospital, Albert Schweitzerlaan 31, 7334 DZ Apeldoorn, The Netherlands; 2 Julius Center for Health Sciences and Primary Care, University Medical Center, Utrecht Heidelberglaan 100, 3584 CX Utrecht, The Netherlands; 3 Department of Intensive Care, Academic Medical Center, Amsterdam, Meibergdreef 9, 1105 AZ, Amsterdam, The Netherlands

## Abstract

**Introduction:**

Acute kidney injury (AKI) is a serious complication in critically ill patients admitted to the Intensive Care Unit (ICU). We hypothesized that ICU survivors with AKI would have a worse health-related quality of life (HRQOL) outcome than ICU survivors without AKI.

**Methods:**

We performed a long-term prospective observational study. Patients admitted for > 48 hours in a medical-surgical ICU were included and divided in two groups: patients who fulfilled RIFLE criteria for AKI and patients without AKI. We used the Short-Form 36 to evaluate HRQOL before admission (by proxy within 48 hours after admission of the patient), at ICU discharge, hospital discharge, 3 and 6 months following ICU discharge (all by patients). Recovery in HRQOL from ICU-admission onwards was assessed using linear mixed modelling.

**Results:**

Between September 2000 and January 2007 all admissions were screened for study participation. We included a total of 749 patients. At six months after ICU discharge 73 patients with AKI and 325 patients without AKI could be evaluated. In survivors with and without AKI, the pre-admission HRQOL (by proxy) and at six months after ICU discharge was significantly lower compared with an age matched general population. Most SF-36 dimensions changed significantly over time from ICU discharge. Change over time of HRQOL between the different AKI Rifle classes (Risk, Injury, Failure) showed no significant differences. At ICU discharge, scores were lowest in the group with AKI compared with the group without AKI for the physical functioning, role-physical and general health dimensions. However, there were almost no differences in HRQOL between both groups at six months.

**Conclusions:**

The pre-admission HRQOL (by proxy) of AKI survivors was significantly lower in two dimensions compared with the age matched general population. Six months after ICU discharge survivors with and without AKI showed an almost similar HRQOL. However, compared with the general population with a similar age, HRQOL was poorer in both groups.

## Introduction

Acute kidney injury (AKI) is a common finding among patients in the intensive care unit (ICU) [1] and occurs in at least 35% to 70% of ICU admissions and approximately 10% will require renal replacement therapy (RRT). AKI mortality is related to severity of injury with a high mortality rate despite the advances in RRT [2]. Furthermore, AKI is frequently a manifestation of multiple organ dysfunction syndrome, and this in itself may explain the high mortality [3]. The cost-effectiveness and rationale both for health-related quality of life (HRQOL) and long-term mortality after initial recovery of ICU patients with AKI have been challenged [4]. An important aspect in this discussion is the HRQOL of patients who have survived AKI and subsequently require RRT in the ICU. Several studies reported that the HRQOL of patients with AKI and treated with RRT in the ICU is impaired [2,3,5-8]. Important shortcomings of many studies in patients with AKI are not only the lack of knowledge regarding the HRQOL before the AKI and before the ICU stay but also long-term outcomes in terms of mortality and HRQOL. Other studies showed that many patients requiring ICU admission have a lower HRQOL before hospital admission [9-11]. Therefore, the first aims of our study were to measure HRQOL before ICU admission and to assess the impact of ICU stay by following the evolution of HRQOL in surviving patients with AKI and those without AKI, up to 6 months after ICU discharge. The second aims were to compare the HRQOL of the patients with AKI with that of patients without AKI and to compare both groups with an age-matched general population. We hypothesized that survivors of AKI would have a worse HRQOL outcome than ICU survivors without AKI.

## Materials and methods

**Figure 1:**
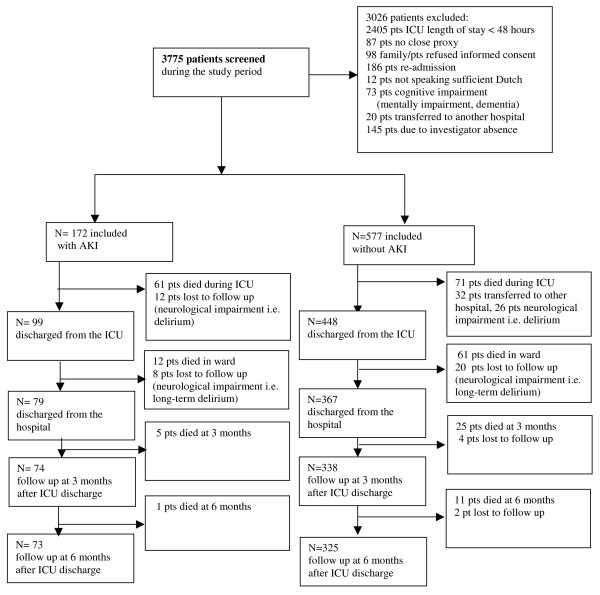
**Flow diagram of the patients screened and included in the study**. AKI, acute kidney injury; ICU, intensive care unit; Pts, patients.

We performed a long-term prospective observational study in a 10-bed closed-format (intensivist led) mixed medical-surgical ICU of Gelre Hospital, a 654-bed university-affiliated teaching hospital in Apeldoorn, The Netherlands. Between September 2000 and January 2007, all admissions were screened for study participation (Figure 1). The local ethics committee approved the study. Informed consent was given by a proxy and, as soon as possible, by the patients themselves. We classified the patients of the AKI group according to the maximum RIFLE (Risk, Injury, Failure, Loss, and End-stage kidney disease) class [12] during their ICU stay and according to urine output data. RIFLE was the current choice when the study was completed. As baseline creatinine, the lowest creatinine value measured in the 3 months before the patient's ICU admission (87.2% of the cases) or creatinine calculated from the MDRD (Modification in Diet in Renal Disease) was used [13]. Patients with pre-existing end-stage renal disease were excluded. Patients with an impaired level of self-awareness or without the ability to communicate adequately at any time during the study were also excluded. We evaluated HRQOL before admission (proxies), at ICU discharge (patients), at hospital discharge (patients), and at 3 and 6 months (patients) after ICU discharge. AKI patients and non-AKI patients admitted for more than 48 hours were included in the study. We included only patients with an ICU stay of more than 48 hours because we aimed to evaluate the sickest patients, hypothesizing that the impact of ICU stay on HRQOL would be most prominent. Patients' demographic data and severity of illness (Acute Physiology and Chronic Health Evaluation II, or APACHE II) [14] were also collected.

### Health-related quality of life measurement

The Short-Form 36 (SF-36 version 1) [15], a widely used generic standardized health status questionnaire, was used to measure HRQOL. This measurement contains eight multi-item dimensions: physical functioning (PF), role limitation due to physical problems (RP), bodily pain (BP), general health (GH), vitality (VT), social functioning (SF), role limitation due to emotional problems (RE), and mental health (MH). Computation of domain scores was performed according to predefined guidelines [16]. Higher scores represent better functioning, and the range of scores was from 0 to 100. Furthermore, scores were aggregated to summary measures representing a physical health summary score (physical component score (PCS), mainly reflecting physical functioning, physical role, pain, and general health) and a mental health summary score (mental component score (MCS), mainly reflecting vitality, social functioning, emotional role, and mental health) [17]. Population scores on PCS and MCS have been standardized on a population mean of 50 and a standard deviation of 10 [18]. The SF-36 was validated in primary care for members of the general population [18,19] and for assessing HRQOL following critical illness [20,21]. Translation, validation, and norming of the Dutch language version of the SF-36 health questionnaire were evaluated in 1998 in community and chronic disease populations [22].

As most of the patients are not able to complete a questionnaire at the time of admission, proxies have to be used frequently as a surrogate approach. The use of proxies to assess the patients' HRQOL was validated in earlier studies by our group by using the SF-36 [23] and the Academic Medical Center Linear Disability score (ALDS) measuring physical reserve [24]. Proxies had to be in close contact with the patient on a regular basis. When necessary, instructions and an explanation of the questionnaire were given. Proxies were asked to answer on behalf of the patient and mark the statement that best described the patient's state of health in the last 4 weeks prior to the admission.

The first SF-36 questionnaire was completed within 48 hours of admission by using the standard time frame of 4 weeks. At the time of ICU discharge and hospital discharge, the patients were specifically asked to score their HRQOL according to their current situation. During hospital admission, patients completed the questionnaire by personal interview. All interviews were performed by the same investigator (JGMH). After hospital discharge, the questionnaire was completed by personal interview or was conducted by telephone. Whenever it was necessary to do so, the investigator (JGMH) visited the patients at home. The average time required to complete the questionnaire was 15 to 20 minutes. Furthermore, we compared HRQOL before ICU admission and 6 months after ICU discharge with those of the 60- to 70-year-old age group of the general Dutch population [22] and used the first question of the SF-36 as a measure of perceived overall health status. This is the single-item question pertaining to general health status: 'In general, would you say your health is excellent, very good, good, fair, or poor?'

### Renal replacement therapy (CVVH)

During the study period, the hemofiltration protocol and machine (Multifiltrate machine (Fresenius Medical Care, Homburg, Germany) were not changed. Continuous venovenous hemofiltration (CVVH) was performed by using an AV600S filter (Fresenius Medical Care). A blood flow of 200 to 300 mL/minute was used, and the substitution flow was set at 35 mL/kg per hour. Anticoagulation was achieved by intravenous infusion of citrate into the afferent line of the CVVH machine in accordance with a standard ICU protocol. No bolus of anticoagulation was given. Two kinds of substitution fluids were used, depending on base deficit: one bicarbonate-buffered (Multibic; Medical Care, Homburg, Germany) and one buffer-free (HF281; Medical Care).

### Statistical analysis

As we aimed to assess how patients improved after ICU discharge, we chose to analyze changes over time from ICU discharge by using a linear mixed model for each dimension of the SF-36 and by using the pre-ICU score as a covariate [25]. The main advantage of such a model is that each measurement of each subject is used, regardless of time of drop-out (like mortality). These models are less biased than complete-case analyses, as the 'worse' patients who eventually drop out of the study are included as much as possible in the estimations. The improvement from ICU discharge is estimated by using data obtained from patients; the proxy assessment is used only to correct for differences in pre-ICU HRQOL between patients. We made the following choices in the linear mixed model: a random intercept model, in which patients were included as a random effect; fixed effects included time, pre-ICU score, APACHE II score, age, AKI/non-AKI, and the interaction between time and AKI/non-AKI (to check whether both groups showed a different pattern of change over time); the final estimation method was restricted maximum likelihood. The assumption of normality of the residuals was assessed by a Q-Q plot. Estimates of domain scores at different time points are presented with 95% confidence intervals. To present the simplest possible mode, we tested whether random slopes needed to be included in the model. We chose to report the models with random slopes for time, as these were significantly better than models without random slopes in all domains. We also analyzed the change over time from ICU discharge for the different RIFLE criteria groups (Risk, Injury, and Failure) within the AKI group and the change over time of patients treated with CVVH within the AKI group. For the comparison of pre-admission versus 6 months, we could not use the linear mixed model, as the pre-admission score was included in that model as a covariate. Therefore, we performed an analysis of covariance (ANCOVA) (that is, a general linear model) with Bonferroni correction (significance level *P *< 0.05) to detect differences in the SF-36 scores at admission between survivors and non-survivors and to assess changes between pre-ICU and 6 months after ICU discharge (repeated measures ANCOVA). A statistical adjustment was made for age, sex, and APACHE II score by including these variables as covariates. SF-36 dimensions of survivors were compared with normative data from the 60- to 70-year-old age group from the Dutch normal population [22] by using the one-sample *t *test. The significance level was adjusted by Bonferroni correction according to the number of related tests conducted. To examine the relative magnitude of changes over time and between groups, effect sizes were used on the basis of the mean change found in a variable divided by the baseline standard deviation. Effect sizes estimate whether particular changes in health status are relevant. In keeping with Cohen, effect sizes of at least 0.20, at least 0.50, and more than 0.80 were considered small, medium, and large changes, respectively [26]. To illustrate the course of HRQOL over time, we plotted raw (uncorrected) data. Groups were defined on the length of follow-up (that is, ranging from only pre-ICU to 6 months after discharge). Chi-squared tests were used to assess the demographic differences between ICU survivors and ICU non-survivors. Data were analyzed by using the Statistical Package for the Social Sciences (version 17; SPSS Inc., Chicago IL, USA) and the Linear mixed-effects models (MIXED) procedure. All data are expressed as mean ± standard deviation where appropriate unless otherwise indicated.

## Results

During the study period, 3,775 patients were screened for study participation. We included a total of 749 patients (Figure 1): 172 with AKI (61.6% were men and 38.4% were women) and 577 without AKI (60.5% were men and 39.5% were women) (Table 1). A baseline HRQOL was obtained from all patients who were evaluated in the final analysis (*n *= 749). Of the patients with/without AKI, HRQOL was measured at ICU discharge (*n *= 99/448), at hospital discharge (*n *= 79/367), and at 3 months (*n *= 74/338) and 6 months (*n *= 73/325). Lost to follow-up were 20/26 patients (mental impairment or long-term delirium) (Figure 1). The demographic and clinical characteristics of the patients lost to follow-up did not differ from those of the group analyzed in the study (data not shown).

**Table 1:** Demographic and clinical characteristics of patients with acute kidney injury (AKI) and those without AKI

	Total group with AKI	Total AKI group (RIFLE criteria) *n *= 172			Total group without AKI	Survivors at 6 months with AKI	Survivors at 6 months without AKI	Differences, total group 172/577	Differences, survivors 73/325
		**Risk**	**Injury**	**Failure**					

Number (percentage)	172	52 (30.2)	53 (30.8)	67 (39.0)	577	73	325	*P *value	*P *value

Age, years	70 (60-77)	70 (64-78)	72 (62.5-80.5)	66 (54-74)	71 (63-77)	67 (54.5-74.0)	69 (61.3-76)	0.898	0.424

Males, number (percentage)	106 (61.6)	39 (22.7)	31 (36.0)	36 20.9)	350 (60.5)	44 (60.3)	190 (58.6)	< 0.001	< 0.001

Females, number (percentage)	66 (38.4)	13 (7.6)	22 (12.8)	31 (36.0)	227 (39.5)	29 (39.7)	134 (41.4)	< 0.001	< 0.001

APACHE II score, points	21 (18-26)	21 (18-26)	21 (19-24.5)	22 (18-27)	17.5 (14-22)	21 (18-26)	17 (13-21)	< 0.001	< 0.001

ICU length of stay, days	13.5 (6-27)	16 (8.2-25.7)	11 (6-22)	12 (6-28)	7 (5-13)	10 (6-18)	7 (5-12)	< 0.001	0.002

Hospital length of stay, days	27 (15.2-52)	22.5 (14.5-55)	24 (13-54)	30 (17-63)	22 (13-37)	35 (20-61.5)	26 (16-41.8)	0.023	0.058

Ventilation, days	10 (3.3-20.7)	15 (5.3-21.5)	9 (3.5-16.5)	9 (2-24)	5 (3-10)	6 (2-13.5)	5 (3-8.7)	< 0.001	0.002

Diagnostic groups, number (percentage)									

Cardiovascular pathology	73 (42.4)	20 (20.8)	24 (12.5)	29 (15.1)	111 (19.2)	33 (45.2)	64 (19.8)	0.006	0.02

Respiratory pathology	48 (27.9)	18 (9.4)	11 (5.7)	19 (9.9)	196 (34.0)	20 (27.4)	97 (29.9)	< 0.001	< 0.001

Gastrointestinal pathology	45 (26.2)	12 (6.3)	16 (8.3)	17 (8.9)	214 (37.1)	19 (26.0)	139 (42.9)	< 0.001	< 0.001

Neurological pathology	2 (1.2)	1 (0.51)	-	1 (0.51)	28 (4.9)	-	7 (2.2)	-	-

Trauma	1 (0.6)	-	1 (0.51)	-	22 (3.8)	-	14 (4.3)	-	-

Others	3 (1.7)	1 (0.51)	1 (0.51)	1 (0.51)	6 (1.0)	1 (1.4)	3 (0.9)	0.317	0.317

Type of admission, number (percentage)									

Non-surgical	110 (64.0)	34 (19.8)	28 (16.3)	48 (27.9)	305 (52.9)	48 (65.8)	138 (42.6)	< 0.001	< 0.001

Emergency surgical	50 (29.1)	15 (8.7)	23 (13.4)	12 (7.0)	207 (35.9)	20 (27.4)	138 (42.6)	< 0.001	< 0.001

Elective surgical	12 (7.0)	3 (1.7)	2 (1.2)	7 (4.1)	65 (11.3)	5 (6.8)	48 (14.8)	< 0.001	< 0.001

Type of proxy, number (percentage)									

Spouse	97 (56.4)	26 (15.3)	35 (20.6)	36 (21.2)	412 (71.4)	51 (69.9)	257 (79.3)	< 0.001	< 0.001

Children	72 (41.9)	30 (17.6)	30 (17.6)	10 (5.9)	157 (27.2)	22 (30.1)	59 (18.2)	< 0.001	< 0.001

Brother/Sister	3 (1.7)	1 (0.6)	2 (1.2)	-	8 (1.4)	-	8 (2.5)	-	-

Elective surgical: intensive care unit (ICU) admission was planned within a 24-hour period before surgery. Emergency surgical: unplanned surgery. Non-surgical: all other admissions. Values are presented as median and interquartile range (P_25_-P_75_) unless stated otherwise. APACHE II, Acute Physiology and Chronic Health Evaluation II; RIFLE, Risk, Injury, Failure, Loss, and End-stage kidney disease.

Among 172 patients with AKI, 30.2% were classified according the RIFLE criteria as risk, 30.8% as injury, and 39% as failure. Of the AKI group, 113 patients (65.7%) were treated with CVVH; of the non-AKI group, 13 patients (2.3%) were treated with CVVH. Mortality rates at 6 months were 46.5% in the group with AKI and 29.1% in the group without AKI (Table 2). ICU and hospital lengths of stay and ventilation days were significantly higher in the total group with AKI compared with the total group without AKI. Demographic and clinical characteristics of all patients are shown in Tables 1 and 2.

**Table 2:** Mortality and Risk, Injury and Failure of Kidney function (RIFLE) criteria

	Total group with AKI	Total AKI group (RIFLE criteria) *n *= 172			Total group without AKI	Survivors at 6 months with AKI	Survivors at 6 months without AKI	Differences, total group 172/577	Differences, survivors 73/325
		**Risk**	**Injury**	**Failure**					

Number	172	52 (30.2)	53 (30.8)	67 (39.0)	577	73	325	*P *value	*P *value

Mortality, number (percentage)	80 (46.5)	30 (37.5)	23 (28.8)	27 (33.8)	168 (29.1)	-	-	< 0.001	-

RIFLE, number (percentage) Risk					-	16 (21.9)	-		

Injury						24 (32.9)			

Failure						33 (45.2)			

CVVH, number (percentage)	113 (65.7)	31 (27.4)	32 (28.3)	50 (44.2)	13 (2.3)	39 (53.4)	8 (2.5)	< 0.001	0.798

CVVH, days	7 (3-12.5)	6 (4-11)	7 (3-11)	7 (2-15)	2 (1.5-3.0)	4 (2-12)	3 (1.3-4.5)	0.567	0.757

Chronic intermittent hemo-dialysis	15	-	-	-	-	-	-	-	-

Renal transplant	2	-	-	-	-	-	-	-	-

CVVH, continuous venovenous hemofiltration; RIFLE, Risk, Injury, Failure, Loss, and End-stage kidney disease.

### Patients with AKI and those without AKI

#### Changes over time in patients with AKI and those without AKI

**Figure 2:**
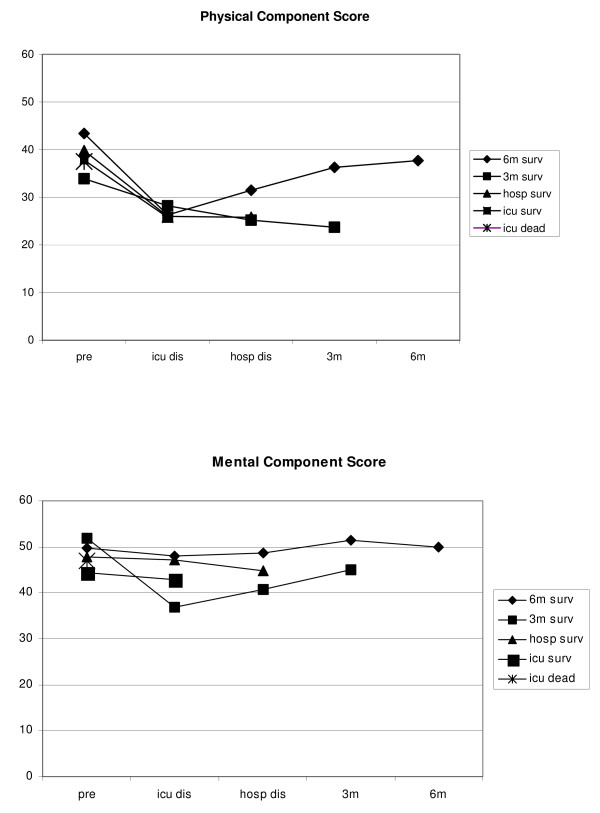
**Course of physical component score and mental component score over time with different survival time of patients with acute kidney injury**. 3 m, 3 months after intensive care unit (ICU) discharge; 6 m, 6 months after ICU discharge; 3 m surv, survivors 3 months after ICU discharge; 6 m surv, survivors 6 months after ICU discharge; hosp dis, hospital discharge; hosp surv, hospital survivors; ICU dead, dead at ICU discharge; ICU dis, ICU discharge; ICU surv, survivors at ICU discharge; Pre, before ICU admission.

Most SF-36 dimensions, except for bodily pain and role-emotional, changed significantly over time from ICU discharge (Additional file 1). Pre-ICU HRQOL score was a significant predictor of change but not APACHE II score, AKI RIFLE classes, or treatment with CVVH within the AKI group. Change over time of HRQOL between the different AKI Rifle classes (Risk, Injury, and Failure) showed no significant differences. Age was a significant predictor of change in the physical functioning dimension only. At ICU discharge, scores were lowest in the group with AKI compared with the group without AKI for the physical functioning, role-physical, and general health dimensions. In both groups, physical functioning was far lower than mental functioning. Surprisingly, bodily pain had a high (that is, positive) score. The course of HRQOL over time of patients with AKI is illustrated in Figure 2 as uncorrected values (that is, not derived from the linear mixed model).

#### Comparison of pre-admission (by proxy) and 6-month HRQOL of survivors with AKI and those without AKI

Pre-admission HRQOL showed no significant differences in any SF-36 dimensions between survivors with AKI and those without AKI. At 6 months, the mean scores of the survivors with AKI were significantly lower in two dimensions - vitality (*P *= 0.016; effect size 0.25) and general health (*P *= 0.039; effect size 0.22) - compared with the survivors without AKI.

### Patients with AKI

#### Comparison of AKI survivors' pre-admission HRQOL (by proxy) with that of the general population

**Figure 3:**
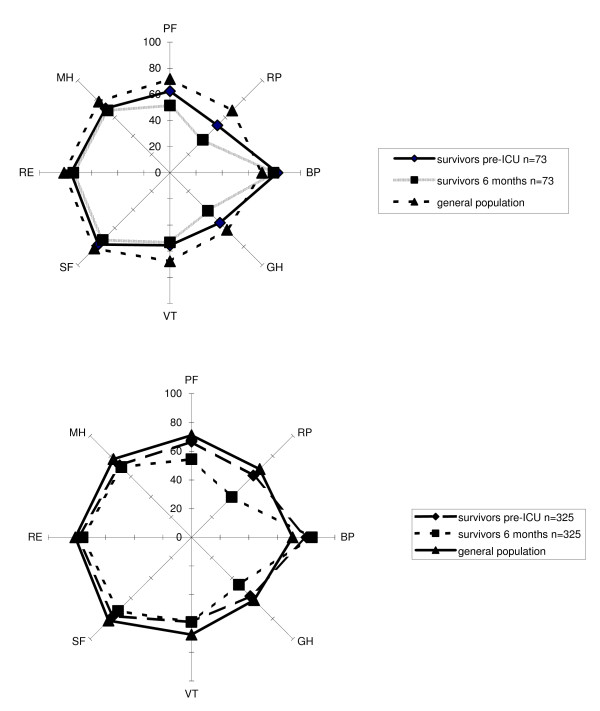
**Comparison of survivors with acute kidney injury (AKI) (*n *= 73) and those without AKI (*n *= 325) before intensive care unit (ICU) admission, 6 months after ICU discharge, and the general Dutch population**. BP, bodily pain; GH, general health; MH, mental health; PF, physical functioning; RE, role limitation due to emotional problems; RP, role limitation due to physical problems; SF, social functioning; VT, vitality.

The pre-admission HRQOL of AKI survivors was significantly lower in two dimensions - vitality (*P *< 0.001) and mental health (*P *< 0.001) - when compared with an age-matched general population (Table 3 and Figure 3). The significant difference of the bodily pain dimension was based on a higher mean pre-admission score compared with the general population.

**Table 3:** Health-related quality of life in surviving patients with acute kidney injury and comparison with general population

SF-36 dimensions	Pre-ICU, all patients	Pre-ICU, survivors (6 months)	ICU discharge	Hospital discharge	3 months after ICU discharge	6 months after ICU discharge	General population (61- to 70-year-old age group)	Differences between pre-ICU survivors (*n *= 73) and non-survivors (*n *= 80)^a^		Differences between pre-ICU survivors and general population^b^		Differences between pre-ICU (*n *= 73) and 6 months after ICU discharge (*n *= 73)^c^		Differences between 6 months after ICU discharge and general population^b^	
Number	172	73	99	79	74	73		*P *value	Effect sizes	*P *value	Effect sizes	Wilks's lambda	Effect sizes	*P *value	Effect sizes

PCS	40.6 ± 12.6	42.3 ± 12.7	26.3 ± 5.9	31.2 ± 7.6	35.9 ± 10.8	37.1 ± 11.3	-	0.158	0.39	-	-	0.001^d^	0.46	-	-

MCS	47.9 ± 10.6	49.7 ± 9.9	46.3 ± 9.4	47.5 ± 9.8	50.8 ± 9.2	49.9 ± 9.1	-	0.051	0.27	-	-	0.924	0.02	-	-

PF	58.7 ± 33.9	62.5 ± 34.2	6.01 ± 12.8	25.5 ± 22.9	48.6 ± 31.4	51.5 ± 32.9	71.7 ± 25.6	0.450	0.28	0.025	0.36	0.014^d^	0.32	< 0.001^d^	0.61

RP	46.1 ± 47.1	51.4 ± 48.9	13.1 ± 31.2	14.2 ± 33.2	26.7 ± 41.0	35.6 ± 42.1	67.3 ± 40.9	0.144	0.32	0.007	0.39	0.027^d^	0.32	< 0.001 ^d^	0.75

BP	78.0 ± 25.6	82.4 ± 25.2	79.0 ± 22.3	83.1 ± 21.3	82.6 ± 24.3	79.4 ± 24.7	70.5 ± 24.6	0.029^d^	0.38	< 0.001^d^	0.48	0.485	0.12	0.003^d^	0.36

GH	48.9 ± 29.3	54.0 ± 29.3	29.0 ± 18.3	36.4 ± 21.5	42.1 ± 22.7	41.2 ± 21.2	61.7 ± 20.2	0.094	0.37	0.028	0.36	0.001^d^	0.44	< 0.001^d^	0.96

VT	52.9 ± 25.2	55.5 ± 27.5	31.4 ± 15.3	44.2 ± 18.3	54.7 ± 20.5	53.3 ± 22.3	67.7 ± 19.6	0.336	0.20	< 0.001^d^	0.52	0.993	0.07	< 0.001^d^	0.64

SF	72.0 ± 24.3	77.6 ± 23.7	49.2 ± 22.9	58.2 ± 23.4	68.7 ± 25.4	72.9 ± 24.6	82.0 ± 24.6	0.014^d^	0.44	0.115	0.19	0.202	0.19	0.002^d^	0.37

RE	70.3 ± 42.3	75.8 ± 40.6	67.7 ± 42.4	62.0 ± 47.1	80.6 ± 37.8	73.9 ± 42.0	81.1 ± 35.0	0.166	0.25	0.268	0.04	0.948	0.04	0.152	0.17

MH	66.4 ± 17.3	69.7 ± 16.1	56.0 ± 11.2	62.3 ± 13.3	67.4 ± 14.0	67.3 ± 13.2	76.9 ± 17.9	0.021^d^	0.40	< 0.001^d^	0.55	0.246	0.15	< 0.001^d^	0.54

Effect sizes of at least 0.20, at least 0.50, and greater than 0.80 correspond to small, medium, and large, respectively. Values are presented as mean ± standard deviation (SD). ^a^Univariate analysis of variance with Bonferroni correction *P *< 0.05 significant. ^b^One-sample *t *test. ^c^General linear model repeated measures with Bonferroni correction *P *< 0.05 significant. ^d^*P *value significant after Bonferroni correction (*P *0.05/10 = *P *= 0.005 = significant). SF-36 (Medical Outcomes Study 36-item Short-Form) dimension scores range from 0 to 100. Physical component score (PCS) and mental component score (MCS) are converted to mean 50 (SD 10). BP, bodily pain; GH, general health; MH, mental health; PF, physical functioning; RE, role limitation due to emotional problems; RP, role limitation due to physical problems; SF, social functioning; VT, vitality.

#### Comparison of AKI survivors' pre-admission HRQOL with that of non-survivors (by proxy)

Pre-admission HRQOL of AKI non-survivors was significantly lower in three dimensions - social functioning (*P *= 0.014), mental health (*P *= 0.021), and bodily pain (*P *= 0.029) - compared with that of AKI survivors (Table 3).

#### Comparison of AKI survivors' pre-admission HRQOL (by proxy) with HRQOL at 6 months

Six months after ICU discharge, four dimensions - physical functioning (*P *= 0.014), role-physical (*P *= 0.027), general health (*P *= 0.001), and the PCS (*P *< 0.001) - were still significantly lower compared with their pre-admission levels (*n *= 73) in survivors with AKI (Table 3).

#### Comparison of AKI survivors' 6-month HRQOL with that of the general population

The 6-month HRQOL of AKI survivors was significantly lower in all dimensions except role-emotional (*P *= 0.152). The significant difference of the bodily pain dimension was based on a higher mean 6-month score compared with the general population (Table 3 and Figure 3).

### Patients without AKI

#### Comparison of pre-admission HRQOL (by proxy) of survivors without AKI with that of the general population

The pre-admission HRQOL of survivors without AKI was significantly lower in three dimensions - vitality (*P *< 0.001), mental health (*P *< 0.001), and social functioning (*P *= 0.001) - when compared with the age-matched general population (Table 4 and Figure 3). The significant difference of the bodily pain dimension was based on a higher mean pre-admission score compared with the general population.

**Table 4:** Health-related quality of life in surviving patients without acute kidney injury and comparison with general population

	Pre-ICU, all patients	Pre-ICU, survivors (6 months)	ICU discharge	Hospital discharge	3 months after ICU discharge	6 months after ICU discharge	General population (61- to 70-year-old age group)	Differences between pre-ICU survivors (*n *= 325) and pre-ICU non-survivors (*n *= 168)^a^		Differences between pre-ICU survivors and general population^b^		Differences between pre-ICU (*n *= 325) and 6 months after ICU discharge (*n *= 325)^c^		Differences between 6 months after ICU discharge and general population^b^	
Number	577	325	448	367	338	325		*P *value	Effect sizes	*P *value	Effect sizes	Wilks's lambda	Effect sizes	*P *value	Effect sizes

PCS	41.5 ± 13.6	44.0 ± 12.9	26.6 ± 6.5	32.5 ± 9.2	36.8 ± 10.5	39.1 ± 11.1	-	< 0.001^d^	0.57	-	-	< 0.001^d^	0.38	-	-

MCS	48.5 ± 10.3	50.1 ± 9.9	46.1 ± 9.3	47.9 ± 9.5	49.7 ± 10.9	50.7 ± 10.9	-	< 0.001^d^	0.35	-	-	0.241	0.06	-	-

PF	59.6 ± 34.6	66.6 ± 33.2	6.4 ± 12.9	30.8 ± 26.4	49.0 ± 32.4	54.3 ± 31.8	71.7 ± 25.6	< 0.001^d^	0.59	0.006	0.20	< 0.001^d^	0.37	< 0.001^d^	0.68

RP	52.5 ± 47.4	60.9 ± 46.7	14.4 ± 32.5	19.6 ± 35.7	28.9 ± 40.5	39.7 ± 45.6	67.3 ± 40.9	< 0.001^d^	0.54	0.014	0.16	< 0.001^d^	0.45	< 0.001^d^	0.67

BP	79.0 ± 26.9	80.2 ± 27.3	75.1 ± 25.0	82.1 ± 22.8	80.7 ± 24.0	84.0 ± 22.2	70.5 ± 24.6	0.238	0.14	< 0.001^d^	0.39	0.033^d^	0.14	< 0.001^d^	0.55

GH	50.9 ± 30.3	57.9 ± 28.9	31.1 ± 19.6	38.8 ± 27.4	44.7 ± 26.4	46.8 ± 24.9	61.7 ± 20.2	< 0.001^d^	0.57	0.019	0.19	< 0.001^d^	0.38	< 0.001^d^	0.74

VT	53.6 ± 24.1	58.9 ± 24.2	32.7 ± 15.9	44.9 ± 18.2	55.9 ± 22.8	58.9 ± 21.7	67.7 ± 19.6	< 0.001^d^	0.59	< 0.001^d^	0.45	0.610	0.10	< 0.001^d^	0.36

SF	71.8 ± 24.7	77.5 ± 24.6	52.1 ± 22.5	60.4 ± 24.8	69.1 ± 27.8	72.5 ± 24.8	82.0 ± 24.6	< 0.001^d^	0.56	0.001^d^	0.18	0.011^d^	0.20	< 0.001^d^	0.39

RE	74.8 ± 40.9	79.3 ± 39.0	60.9 ± 44.3	68.2 ± 45.6	70.0 ± 43.2	75.9 ± 40.9	81.1 ± 35.0	0.010^d^	0.29	0.412	0.05	0.383	0.08	0.027	0.15

MH	67.5 ± 16.7	70.9 ± 15.9	56.9 ± 11.5	63.1 ± 13.0	67.6 ± 16.1	69.2 ± 21.5	76.9 ± 17.9	< 0.001^d^	0.50	< 0.001^d^	0.34	0.461	0.08	< 0.001^d^	0.43

Effect sizes of at least 0.20, at least 0.50, and greater than 0.80 correspond to small, medium, and large, respectively. Values are presented as mean ± standard deviation (SD). ^a^Univariate analysis of variance with Bonferroni correction *P *< 0.05 significant. ^b^One-sample *t *test. ^c^General linear model repeated measures with Bonferroni correction *P *< 0.05 significant. ^d^*P *value significant after Bonferroni correction (*P *0.05/10 = *P *= 0.005 = significant). SF-36 (Medical Outcomes Study 36-item Short-Form) dimension scores range from 0 to 100. Physical component score (PCS) and mental component score (MCS) are converted to mean 50 (SD 10). BP, bodily pain; GH, general health; ICU, intensive care unit; MH, mental health; PF, physical functioning; RE, role limitation due to emotional problems; RP, role limitation due to physical problems; SF, social functioning; VT, vitality.

#### Comparison of pre-admission HRQOL (by proxy) in survivors with that of non-survivors without AKI

Pre-admission HRQOL was significantly lower in all dimensions of the SF-36, except bodily pain, of non-survivors without AKI compared with the survivors without AKI (Table 4).

#### Comparison of pre-admission HRQOL (by proxy) with HRQOL at 6 months in survivors without AKI

In the patients without AKI, five dimensions were significantly lower at 6 months compared with their pre-admission HRQOL: physical functioning (*P *< 0.001), role-physical (*P *< 0.001), general health (*P *< 0.001), social functioning (*P *= 0.011), and the PCS (*P *< 0.001). The significant difference of the bodily pain dimension was based on a higher mean 6-month score compared with pre-admission (Table 4).

#### Comparison of 6-month HRQOL of survivors without AKI with that of the general population

The 6-month HRQOL of survivors without AKI was significantly lower in all dimensions except role-emotional (*P *= 0.027). The significant difference of the bodily pain dimension was based on a higher mean 6-month score compared with the general population (Table 4 and Figure 3).

## Discussion

The main finding of the present study is that survivors with AKI and those without AKI showed almost no significant differences between the HRQOL 6 months after ICU discharge, suggesting that AKI survivors do not have a perceived lower HRQOL outcome than ICU survivors without AKI. Our study results showed that, in survivors with AKI and those without AKI, the pre-admission HRQOL (by proxy) was significantly lower compared with the age-matched general population. At 6 months after ICU discharge, HRQOL in survivors of both groups was lower than pre-ICU admission HRQOL. Furthermore, HRQOL of survivors of both groups at 6 months was far lower than that of the general population. These differences in results at 6 months can be due to the different means of taking measures.

Interestingly, hospital length of stay was longer in the AKI survivors compared with the total AKI group, which was obviously affected by death. Thereby, patients with AKI (total group) had a significantly longer ICU length of stay and ventilation days than patients without AKI (total group), and this may have important implications for resource utilization. In particular, the longer ICU and hospital stays may be associated with a higher rate of transfer to a long-term care or rehabilitation facility after hospital discharge [27,28]. In our study, mortality of the AKI group was high (46.5%). Renal failure severe enough to require RRT has been associated with a high mortality in various studies [29-31] (that is, mortality rates of 55% to 62% at 6 months and 65% at 5 years [3]). In line with our study findings, the majority of deaths occurred during ICU stay. Gopal and colleagues [6] and Morgera and colleagues [7], on the other hand, have reported much higher mortality rates over a similar follow-up time. This is likely due to differences in the severity of illness or due to time bias. Previous studies reported a prevalence of RIFLE-defined AKI at the time of ICU admission of between 22% and 36%, which is comparable to our findings (23%) [1,32]. The total group of included patients in our study showed an AKI risk prevalence comparable to that of a recent study [33], although the AKI injury and failure group were both smaller than in our study. However, we included patients with AKI at the time of ICU admission or during ICU stay, and this may partly explain these differences.

Furthermore, our study revealed that, in survivors with AKI and those without AKI, the pre-admission HRQOL (by proxy) was significantly lower (respectively, three and four dimensions) compared with that of the age-matched general population. These findings are in accord with other studies in which many patients requiring ICU actually have a lower HRQOL before ICU admission [9,34]. An important shortcoming of many studies in the field of long-term prognosis in patients with AKI is the lack of knowledge regarding the HRQOL before ICU stay [35]. When there is no baseline measure of HRQOL, investigators are unable to conclude whether the limitations in HRQOL are due to pre-existing chronic illness or due to the acute condition [8]. However, owing to the patient's condition at admission, assessment of ICU pre-admission scores is rarely possible. Considering the positive results of our earlier validation study [23] and comparable results from other studies [20,36], we chose to use proxies for pre-admission scores. Concerns about proxy estimations of HRQOL in populations with high disease severity [37] are probably based on major differences in timing of assessments of HRQOL: interviewing patients 3 months after ICU discharge and their proxies at study entry. It is likely that the critical illness may influence the patients' retrospective recollection of their previous health (recall bias) and that they may overestimate their previous health.

Interestingly, in our study, survivors with AKI and those without AKI showed no significant differences between the HRQOL 6 months after ICU discharge, except for the vitality and general health dimensions. However, both groups showed a significantly lower HRQOL at 6 months compared with an age-matched general population. Recently, Orwelius and colleagues [38] divided their study group and the reference group into the previously healthy and those having pre-existing disease and reported that a large reduction of HRQOL after ICU is attributable to pre-existing disease. However, the Dutch general population, based on age, gender, and chronic health conditions, does not include a group with no pre-existing disease [22], making it difficult to perform this comparison. Some earlier studies have reported a fairly good HRQOL after AKI [2,3,6]. Korkeila and colleagues [3] reported that, predominantly, overall energy and the physical domain were impaired. However, a limitation of that study is that the patients were not compared with the general population. Most critical care professionals consider the perception of post-discharge HRQOL to be important in their decisions concerning the allocation of intensive care resources [6].

As in our study, Ahlstrom and colleagues [5] investigated the survival and quality of life of patients with AKI/requiring RRT and found also a rather low HRQOL compared with the general population. Comparable findings were reported by Noble and colleagues [8].

Contrary to our findings, other studies found mental health to be comparable and their physical health to be only slightly poorer than that of the general population [2,3]. The results of our study revealed that, at 6 months, physical health was affected more seriously than mental health. These findings concur with those of Delannoy and colleagues [39], who reported that, at 6 months, HRQOL was lower in patients with AKI/RRT than in an age-matched general population. However, in contrast with our study, in the study by Delannoy and colleagues [39] and in the study by Noble and colleagues [8], there was no assessment of HRQOL on ICU admission.

Interestingly, Maynard and colleagues [2] reported that almost all patients, even those with the worst self-reported quality of life and greatest physical disability, considered RRT to have been the right decision. Most survivors valued their lives highly, regardless of their self-reported HRQOL, and in retrospect agreed with the decision to continue aggressive care, including renal replacement therapy [2]. In line with the study by Maynard and colleagues [2], the perceived overall state of HRQOL (using the first question of the SF-36) of the surviving patients with AKI and those without AKI seemed acceptable and showed no significant difference between the two groups. This is also illustrated by the findings of our study that, in contrast to impairment in physical dimensions, perceived mental health seems to be less impaired [8]. Finally, the pre-admission HRQOL of the non-survivors in our study was significantly lower compared with survivors in the group without AKI. Interestingly, in the AKI group, this was the case in only a few dimensions, possibly owing to the smaller group of patients with AKI.

Several limitations to our study should be mentioned. First, we included only patients on their first admission who also stayed in the ICU for more than 48 hours. Therefore, the results are not generalizable to the group of patients with a short ICU stay. Second, as mentioned above, we chose to use proxies for pre-admission scores, instead of a retrospective assessment at ICU discharge. This was done because the critical illness may influence the patients' recollection of their previous health and the approach of using proxies in this setting was validated in an earlier study by our group [23] and by other studies [20,36]. It is known that proxies may differ in their assessment from patients themselves, but our earlier results showed that these differences were small. However, the results between proxy and ICU patient measures should be interpreted with caution.

Third, the presence of delirium could have influenced the response, although we made an effort to identify delirious patients. Finally, we cannot rule out that response shift played a role in our study population (that is, self-evaluation changes resulting from changes in internal standards or values in patients confronted with a life-threatening disease or chronic incurable disease [40]). Social functioning, for instance, could be perceived differently in the clinical setting because of the many visitors in the hospital and cards received.

## Conclusions

Six months after ICU discharge, survivors with AKI and those without AKI showed a nearly similar HRQOL. However, HRQOL is poorer in both groups compared with the general population with a similar age.

## Key messages

• Health-related quality of life (HRQOL) outcome of acute kidney injury (AKI) survivors was nearly similar to that of intensive care unit (ICU) survivors without AKI.

• Patients with AKI and those without AKI demonstrated that recovery of HRQOL was not complete at 6 months after ICU discharge in comparison with their pre-admission HRQOL.

• HRQOL was lower at 6 months after ICU discharge in patients with AKI and those without AKI compared with that of a general population.

## Abbreviations

AKI: acute kidney injury; ANCOVA: analysis of covariance; APACHE II: Acute Physiology and Chronic Health Evaluation II; CVVH: continuous venovenous hemofiltration; HRQOL: health-related quality of life; ICU: intensive care unit; MCS: mental component score; PCS: physical component score; RIFLE: Risk, Injury, Failure, Loss, and End-stage kidney disease; RRT: renal replacement therapy; SF-36: Medical Outcomes Study 36-item Short-Form.

## Competing interests

The authors declare that they have no competing interests.

## Authors' contributions

JGMH performed the study, analyzed and interpreted the data, and drafted the manuscript. HFvS analyzed the data, contributed to the interpretation of the data, and revised the manuscript for important intellectual content. AJPS contributed to the interpretation of the data. JHR conceived of the study and contributed to its design and the interpretation of the data. PES conceived of the study, contributed to the interpretation of the data, and revised the manuscript for important intellectual content. All authors contributed substantially to the manuscript and approved the final version submitted for publication.

## Supplementary Material

Additional file 1 **Estimates of change over time of HRQOL from ICU discharge in the patients with and without AKI**. Changes over time of Short-Form 36 dimensions in patients with and without AKI. Click here for file

## References

[B1] Hoste EA Clermont G Kersten A Venkataraman R Angus DC De Bacquer D Kellum JA RIFLE criteria for acute kidney injury are associated with hospital mortality in critically ill patients: a cohort analysis Crit Care 2006 17 R73 10.1186/cc4915 16696865 PMC1550961

[B2] Maynard SE Whittle J Chelluri L Arnold R Quality of life and dialysis decisions in critically ill patients with acute renal failure Intensive Care Med 2003 17 1589 1593 10.1007/s00134-003-1837-5 12819880

[B3] Korkeila M Ruokonen E Takala J Costs of care, long-term prognosis and quality of life in patients requiring renal replacement therapy during intensive care Intensive Care Med 2000 17 1824 1831 10.1007/s001340000726 11271091

[B4] Hamel MB Phillips RS Davis RB Desbiens N Connors AF Jr Teno JM Wenger N Lynn J Wu AW Fulkerson W Tsevat J Outcomes and cost-effectiveness of initiating dialysis and continuing aggressive care in seriously ill hospitalized adults. SUPPORT Investigators. Study to Understand Prognoses and Preferences for Outcomes and Risks of Treatments Ann Intern Med 1997 17 195 202 9245224 10.7326/0003-4819-127-3-199708010-00003

[B5] Ahlstrom A Tallgren M Peltonen S Rasanen P Pettila V Survival and quality of life of patients requiring acute renal replacement therapy Intensive Care Med 2005 17 1222 1228 10.1007/s00134-005-2681-6 16049711

[B6] Gopal I Bhonagiri S Ronco C Bellomo R Out of hospital outcome and quality of life in survivors of combined acute multiple organ and renal failure treated with continuous venovenous hemofiltration/hemodiafiltration Intensive Care Med 1997 17 766 772 10.1007/s001340050407 9290991

[B7] Morgera S Kraft AK Siebert G Luft FC Neumayer HH Long-term outcomes in acute renal failure patients treated with continuous renal replacement therapies Am J Kidney Dis 2002 17 275 279 10.1053/ajkd.2002.34505 12148099

[B8] Noble JS Simpson K Allison ME Long-term quality of life and hospital mortality in patients treated with intermittent or continuous hemodialysis for acute renal and respiratory failure Ren Fail 2006 17 323 330 10.1080/08860220600591487 16771248

[B9] Graf J Koch M Dujardin R Kersten A Janssens U Health-related quality of life before, 1 month after, and 9 months after intensive care in medical cardiovascular and pulmonary patients Crit Care Med 2003 17 2163 2169 10.1097/01.CCM.0000079607.87009.3A 12973175

[B10] Konopad E Noseworthy TW Johnston R Shustack A Grace M Quality of life measures before and one year after admission to an intensive care unit Crit Care Med 1995 17 1653 1659 10.1097/00003246-199510000-00008 7587229

[B11] Wehler M Geise A Hadzionerovic D Aljukic E Reulbach U Hahn EG Strauss R Health-related quality of life of patients with multiple organ dysfunction: individual changes and comparison with normative population Crit Care Med 2003 17 1094 1101 10.1097/01.CCM.0000059642.97686.8B 12682478

[B12] Bellomo R Ronco C Kellum JA Mehta RL Palevsky P Acute renal failure - definition, outcome measures, animal models, fluid therapy and information technology needs: the Second International Consensus Conference of the Acute Dialysis Quality Initiative (ADQI) Group Crit Care 2004 17 R204 R212 10.1186/cc2872 15312219 PMC522841

[B13] National Kidney Foundation K/DOQI clinical practice guidelines for chronic kidney disease evaluation, classification, and stratification Am J Kidney Dis 2002 17 S1 266 11904577

[B14] Knaus WA Draper EA Wagner DP Zimmerman JE APACHE II: a severity of disease classification system Crit Care Med 1985 17 818 829 10.1097/00003246-198510000-00009 3928249

[B15] Ware JE Jr Sherbourne CD The MOS 36-item short-form health survey (SF-36). I. Conceptual framework and item selection Med Care 1992 17 473 483 10.1097/00005650-199206000-00002 1593914

[B16] Ware JE Health Survey Manual and Interpretation Guide 1993 Boston: Medical Outcomes Trust

[B17] Ware JE Kosinski M Interpreting SF-36 summary health measures: a response Qual Life Res 2001 17 405 413 10.1023/A:1012588218728 11763203

[B18] Brazier JE Harper R Jones NM O'Cathain A Thomas KJ Usherwood T Westlake L Validating the SF-36 health survey questionnaire: new outcome measure for primary care BMJ 1992 17 160 164 10.1136/bmj.305.6846.160 1285753 PMC1883187

[B19] Jenkinson C Coulter A Wright L Short form 36 (SF36) health survey questionnaire: normative data for adults of working age BMJ 1993 17 1437 1440 10.1136/bmj.306.6890.1437 8518639 PMC1677870

[B20] Chrispin PS Scotton H Rogers J Lloyd D Ridley SA Short Form 36 in the intensive care unit: assessment of acceptability, reliability and validity of the questionnaire Anaesthesia 1997 17 15 23 10.1111/j.1365-2044.1997.015-az014.x 9014540

[B21] Heyland DK Hopman W Coo H Tranmer J McColl MA Long-term health-related quality of life in survivors of sepsis. Short Form 36: a valid and reliable measure of health-related quality of life Crit Care Med 2000 17 3599 3605 10.1097/00003246-200011000-00006 11098960

[B22] Aaronson NK Muller M Cohen PD Essink-Bot ML Fekkes M Sanderman R Sprangers MA te Velde A Verrips E Translation, validation, and norming of the Dutch language version of the SF-36 Health Survey in community and chronic disease populations J Clin Epidemiol 1998 17 1055 1068 10.1016/S0895-4356(98)00097-3 9817123

[B23] Hofhuis J Hautvast JL Schrijvers AJ Bakker J Quality of life on admission to the intensive care: can we query the relatives? Intensive Care Med 2003 17 974 979 12734653 10.1007/s00134-003-1763-6

[B24] Hofhuis JG Dijkgraaf MG Hovingh A Braam RL van de Braak L Spronk PE Rommes JH The Academic Medical Center Linear Disability Score for evaluation of physical reserve on admission to the ICU: can we query the relatives? Crit Care 2011 17 R212 10.1186/cc10447 21917138 PMC3334756

[B25] Twisk JWR Applied Longitudinal Data Analysis for Epidemiology 2003 Cambridge: Cambridge University Press

[B26] Cohen J Statistical Power Analysis for the Behavioral Sciences 1988 Hillsdale, NJ: Lawrence Erlbaum Associates

[B27] Bagshaw SM Laupland KB Doig CJ Mortis G Fick GH Mucenski M Godinez-Luna T Svenson LW Rosenal T Prognosis for long-term survival and renal recovery in critically ill patients with severe acute renal failure: a population-based study Crit Care 2005 17 R700 R709 10.1186/cc3879 16280066 PMC1414056

[B28] Chertow GM Christiansen CL Cleary PD Munro C Lazarus JM Prognostic stratification in critically ill patients with acute renal failure requiring dialysis Arch Intern Med 1995 17 1505 1511 10.1001/archinte.1995.00430140075007 7605152

[B29] Bellomo R Farmer M Boyce N Combined acute respiratory and renal failure: management by continuous hemodiafiltration Resuscitation 1994 17 123 131 10.1016/0300-9572(94)90084-1 7846371

[B30] Schaefer JH Jochimsen F Keller F Wegscheider K Distler A Outcome prediction of acute renal failure in medical intensive care Intensive Care Med 1991 17 19 24 10.1007/BF01708404 1903797

[B31] Spiegel DM Ullian ME Zerbe GO Berl T Determinants of survival and recovery in acute renal failure patients dialyzed in intensive-care units Am J Nephrol 1991 17 44 47 10.1159/000168271 2048578

[B32] Bagshaw SM George C Dinu I Bellomo R A multi-centre evaluation of the RIFLE criteria for early acute kidney injury in critically ill patients Nephrol Dial Transplant 2008 17 1203 1210 17962378 10.1093/ndt/gfm744

[B33] Gammelager H Christiansen CF Johansen MB Tonnesen E Jespersen B Sorensen HT One-year mortality among Danish intensive care patients with acute kidney injury: a cohort study Crit Care 2012 17 R124 10.1186/cc11420 22789072 PMC3580703

[B34] Hofhuis JG Spronk PE van Stel HF Schrijvers GJ Rommes JH Bakker J The impact of critical illness on perceived health-related quality of life during ICU treatment, hospital stay, and after hospital discharge: a long-term follow-up study Chest 2008 17 377 385 10.1378/chest.07-1217 17925419

[B35] Druml W Long term prognosis of patients with acute renal failure: is intensive care worth it? Intensive Care Med 2005 17 1145 1147 10.1007/s00134-005-2682-5 16049710

[B36] Rogers J Ridley S Chrispin P Scotton H Lloyd D Reliability of the next of kins' estimates of critically ill patients' quality of life Anaesthesia 1997 17 1137 1143 10.1111/j.1365-2044.1997.240-az0374.x 9485965

[B37] Scales DC Tansey CM Matte A Herridge MS Difference in reported pre-morbid health-related quality of life between ARDS survivors and their substitute decision makers Intensive Care Med 2006 17 1826 1831 10.1007/s00134-006-0333-0 16957904

[B38] Orwelius L Nordlund A Nordlund P Simonsson E Backman C Samuelsson A Sjoberg F Pre-existing disease: the most important factor for health related quality of life long-term after critical illness: a prospective, longitudinal, multicentre trial Crit Care 2010 17 R67 10.1186/cc8967 20398310 PMC2887189

[B39] Delannoy B Floccard B Thiolliere F Kaaki M Badet M Rosselli S Ber CE Saez A Flandreau G Guerin C Six-month outcome in acute kidney injury requiring renal replacement therapy in the ICU: a multicentre prospective study Intensive Care Med 2009 17 1907 1915 10.1007/s00134-009-1588-z 19693486

[B40] Sprangers MA Schwartz CE Integrating response shift into health-related quality of life research: a theoretical model Soc Sci Med 1999 17 1507 1515 10.1016/S0277-9536(99)00045-3 10400253

