# Supplementary material for: The effect of acute kidney injury on long-term health-related quality of life: a prospective follow-up study
Source: Crit Care. 2013 Jan 28;17(1):R17. doi: 10.1186/cc12491 (PMC4057105; doi:10.1186/cc12491)
Supplement: Additional file 1 — Estimates of change over time of HRQOL from ICU discharge in the patients with and without AKI. Changes over time of Short-Form 36 dimensions in patients with and without AKI. [file cc12491-S1.DOC]

**Additional file 1. Estimates of change over time from ICU discharge in the patients with and without AKI***

|  | **ICU-discharge (Intercept)** | **95%CI** | **Change per month** | **95%CI** | **Pre-ICU score #** | **95%CI** | **with AKI+ vs. without AKI ##** | **95%CI** | **Interaction**  **time** | **95%CI** |
| --- | --- | --- | --- | --- | --- | --- | --- | --- | --- | --- |
| PCS | 25.37 | 21.67-29.07 | 0.92 | 0.70-1.14 | 0.19 | 0.13-0.25 | -0.84 | -3.43-1.76 | -0.13 | -0.63-0.37 |
| **MCS** | 36.55 | 30.85-42.24 | 0.34 | 0.13-0.55 | 0.25 | 0.17-0.32 | -0.18 | -2.83-2.48 | -0.05 | -0.53-0.43 |
| **PF** | 33.18 | 18.24-48.17 | 3.29 | 2.70-3.88 | 0.32 | 0.26-0.39 | -5.60 | -12.80-1.60 | 0.33 | -1.02-1.68 |
| **RP** | 4.90 | -12.91-22.70 | 2.99 | 2.05-3.94 | 0.08 | 0.02-0.14 | -3.68 | -14.26-6.89 | 0.02 | -2.14-2.17 |
| **GH** | 35.22 | 23.04-47.39 | 0.93 | 0.38-1.47 | 0.21 | 0.15-0.28 | -1.40 | -8.79-5.99 | -0.46 | -1.71-0.78 |
| **MH** | 46.94 | 38.73-55.15 | 0.86 | 0.47-1.24 | 0.29 | 0.22-0.35 | -0.40 | -4.14-3.34 | -0.26 | -1.12-0.61 |
| **BP** | 64.45 | 52.78-76.11 | 0.18 | -0.27-0.64 | 0.03 | -0.03-0.09 | 3.19 | -2.95-9.33 | -0.88 | -1.92-0.16 |
| **RE** | 56.60 | 37.20-76.00 | 0.89 | -0.07-1.84 | 0.13 | 0.05-0.20 | -2.83 | -15.19-9.53 | 0.72 | -1.46-2.89 |
| **SF** | 41.78 | 28.94-54.61 | 1.60 | 1.09-2.09 | 0.30 | 0.22-0.37 | -1.94 | -8.56-4.68 | 0.14 | -1.01-1.29 |
| **VT** | 42.01 | 31.49-52.52 | 2.03 | 1.61-2.45 | 0.21 | 0.14-0.27 | -0.29 | -5.61-5.04 | -0.74 | -1.68-0.22 |

* linear mixed model with random intercept and random slope (for time).

# Pre-ICU estimate: change in discharge-ICU score (intercept) for one point higher pre-ICU score.

**##** difference in discharge-ICU score (intercept) for patients with AKI versus without AKI

PCS= physical component score; MCS= mental component score; PF= physical functioning; RP= role limitation due to physical problems, GH=general health;

MH=mental health; BP= bodily pain; RE= role limitation due to emotional problems; SF= social functioning; VT= vitality.
